# Setting Mechanical Properties of High Strength Steels for Rapid Hot Forming Processes

**DOI:** 10.3390/ma9040229

**Published:** 2016-03-25

**Authors:** Christian Löbbe, Oliver Hering, Lars Hiegemann, A. Erman Tekkaya

**Affiliations:** Institute of Forming Technology and Lightweight Construction, TU Dortmund University, Baroper Str. 303, Dortmund 44227, Germany; oliver.Hering@iul.tu-dortmund.de (O.H.); Lars.Hiegemann@iul.tu-dortmund.de (L.H.); Erman.Tekkaya@iul.tu-dortmund.de (A.E.T.)

**Keywords:** press hardening, hot forming, progressive die, control of mechanical properties, high strength steel

## Abstract

Hot stamping of sheet metal is an established method for the manufacturing of light weight products with tailored properties. However, the generally-applied continuous roller furnace manifests two crucial disadvantages: the overall process time is long and a local setting of mechanical properties is only feasible through special cooling techniques. Hot forming with rapid heating directly before shaping is a new approach, which not only reduces the thermal intervention in the zones of critical formability and requested properties, but also allows the processing of an advantageous microstructure characterized by less grain growth, additional fractions (e.g., retained austenite), and undissolved carbides. Since the austenitization and homogenization process is strongly dependent on the microstructure constitution, the general applicability for the process relevant parameters is unknown. Thus, different austenitization parameters are analyzed for the conventional high strength steels 22MnB5, Docol 1400M, and DP1000 in respect of the mechanical properties. In order to characterize the resulting microstructure, the light optical and scanning electron microscopy, micro and macro hardness measurements, and the X-ray diffraction are conducted subsequent to tensile tests. The investigation proves not only the feasibility to adjust the strength and ductility flexibly, unique microstructures are also observed and the governing mechanisms are clarified.

## 1. Introduction

Today, hot stamping is established in the automobile industry to comply with demands for safety and lightweight construction through the appropriate setting of mechanical properties [1,2]. The amount of hot stamped parts is continuously rising and accounts for approx. 30% of the body in modern cars [3]. Although tailored mechanical properties (e.g., strength, hardness, and strain at fracture) can be achieved in classical hot stamping by adapted cooling in the tool, the full potential is limited by the applied furnace heating. Rapid hot forming on the basis of high power and process integrated heating methods is an approach to cover outstanding mechanical properties and a more flexible design by forcing dynamic phase transformation effects and the locally-adapted thermal cycle. The one-shot hot stamping by resistance heating before combined forming, cutting, and quenching, shows in an excellent way that both tailored properties and improved part functionality through rapid processing and, thereby, controlled shaping, can be achieved simultaneously [4]. For more complex plates, direct contact heating has been developed, which allows a comparably fast heating and, thus, promotes higher ductility by refined grains [5,6]. Moreover, induction heating is a promising high-performance method with heating rates up to 200 K/s, in which the contactless energy transfer also allows the continuous heating [7]. 

**Figure 1:**
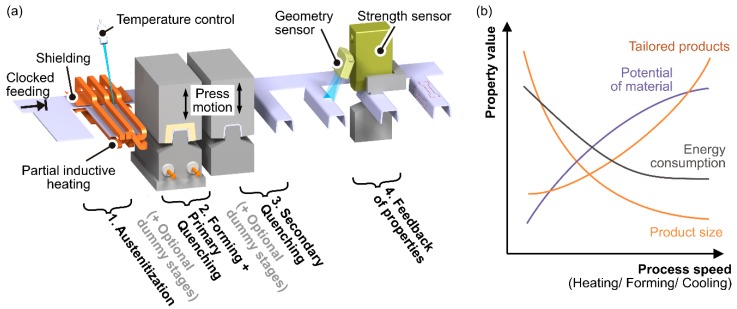
(**a**) Principle of hot stamping in a progressive die; and (**b**) expected characteristic from higher process speed in hot forming processes.

Thus, the proposed approach of hot forming in progressive dies is based on an integrated induction heating in order to apply the benefits of dynamic processing to the large scale production of relatively small and complex formed parts [8]. Figure 1a shows an example process layout, where the local heating occurs directly before forming, the cooling is done by one or more subsequent stations, and a final feedback of mechanical properties can be implemented for a closed loop control. When high cycle frequencies higher than in standard press hardening processes are achieved, not only a larger output is obtained but, also, a higher material potential due to dynamic effects and conditions of imbalance is expected (see Figure 1b). As a side effect, a reduction of energy consumption in consequence of lower heat losses is possible. Nevertheless, the existing “bottleneck” forces a reduction of dimensions when, e.g., the heat transfer is a limiting boundary condition [9].

When the austenitization is conducted within a furnace, the process takes several minutes due to the limited heat exchange on the surface [10]. Furthermore, annealing slightly above the A_c3_ temperature requires a relatively long duration to reach a state of equilibrium for later high quenchability (e.g., 22MnB5, ferritic-pearlitic microstructure: four minutes at 950 °C for tensile strength of 1500 MPa) [10]. Hikida *et al.* [11] found out that, e.g., an initial fracture of cementite and undistributed boron in a press-hardenable steel strongly delays austenitization and homogenization. Thus, the rapid processing is ideally based on a finely dispersed microstructure (e.g., martensitic fractions), so that a major overheating and the risk of grain growth is diminished.

Depending on the chemical composition, the microstructure, and the surface condition, different low-carbon and low-alloyed steels [12], dual-phase steels [13], or full martensitic steels [14] are applicable in a hot stamping process apart from the standard boron manganese steels in order to improve the formability or set tailored properties. Naderi [14] investigated ten low-carbon and low-alloyed steels considering furnace heating and different annealing and cooling parameters. Although high annealing temperatures promote the hardness through grain growth and a diffusionless transformation, a minimum austenitization temperature and duration should be selected in order to avoid a deteriorated ductility. 

In contrast to that, the application of rapid austenitization at very high heating rates offers outstanding material properties through a very short dwell time. For example, the heat treatment of a low-alloyed steel (AISI8620) at an overall duration of less than 10 s allowed the production of a fine microstructure with remaining carbides [15]. The high tensile strength (1600 MPa) is accompanied by a promising strain at fracture (10%), which is promoted by a fine structure, remaining carbides, and a mixture of martensitic and bainitic phases. Azevedo *et al.* [16] found out that this significant fine bainitic structure can be obtained and adjusted by fast heating. In addition, Holzweißig *et al.* analyzed the effect of carbides and a grain refinement by rapid heating to promote more nucleation points for 22MnB5 [5] and later for 30NiCrMnB5-3-3 [17]. The TEM images revealed remaining carbides from the original ferritic-pearlitic microstructure, which were embedded in the martensitic fractions and improved the strength significantly (tensile strength of 1850 MPa, 1000 °C for 10 s). Beyond a high heating rate, Senuma and Takemoto [18] investigated a manganese enriched boron steel (3% Mn) for the fast processing at short term holding. In comparison to the furnace austenitization at 950 °C, the accelerated treatment led to a three times higher absorbable energy in the Charpy impact test due to the significantly refined grains.

In order to unlock the high potential of conventional steels through an accelerated annealing procedure, the processing based on the proposed progressive die technology is investigated for three high strength steels and relevant parameters according to the technology restrictions. Since the objective of the approach is a minimum thermal intervention, the martensitic steels Docol 1400M and DP1000 are studied, besides the hot stamping steel 22MnB5, in a soft and hard condition. In the hard condition, these steels are brittle with a single-digit elongation so that the forming of complex shaped parts is strongly restricted. Thus, new knowledge about the applicability of rapid processing in order to enhance the formability and set tailored properties for an increasingly important lightweight design will be gained [2]. Since the numerous experimental investigations do not cover the thermally-assisted forming of conventionally cold formed steels and, furthermore, do not reveal the ideal austenitization parameters for short term treatment, the expected properties after cooling at tool relevant cooling rates and, in particular, the effect of the interrupted cooling during martensite formation are unknown. Additionally, the application of modern numerical modeling techniques is only possible to a limited extent due to the complex interaction of microconstituents, the local distribution, and the present fractions and their condition. Thus, this paper focuses on both the austenitization and the intermittent cooling under accelerated conditions. In the first step, the effect of different austenitization temperatures *T*_γ_ and dwell times *t*_γ_ on the mechanical properties and the microstructure is experimentally disclosed. On the basis of these findings, ideal austenitization parameters for high strength or high ductility shall be found later. Subsequently, different intermittent quenching strategies are investigated in relation to the resulting microstructure topography and volume fractions. In this context, the stepwise cooling with a holding time during martensite formation is analyzed to retain austenite, which promotes a higher strain combined with a slightly decreased strength [19]. Thus, the objective of this paper is to clarify the governing mechanism, to gain basic knowledge for the application of hot stamping on the basis of the proposed technology, and to determine appropriate parameters for the setting of mechanical properties.

## 2. Experimental Setup

**Figure 2:**
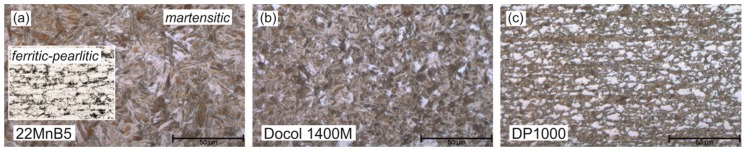
Initial microstructures: (**a**) Ferritic-pearlitic and water quenched martensitic 22MnB5; (**b**) Martensitic Docol 1400M; and (**c**) ferritic-martensitic DP1000.

Table 1 gives the chemical compositions of the uncoated steels 22MnB5, Docol 1400M, and DP1000, which are delivered with the thicknesses 1.5 mm, 1.2 mm, and 1.2 mm. In delivery condition, the 22MnB5 steel from ThyssenKrupp (Dortmund, Germany) manifests approx. 75% ferrite and 25% pearlite, with a measured average grain size of ASTM 10 (see Figure 2a). In order to analyze both ferritic-pearlitic and fully-martensitic microstructures, a conventional furnace annealing and water quenching procedure at 900°C for eight minutes is conducted (prior austenite grain size ~ASTM 7.5). The cold-rolled Docol 1400M and DP1000 steels from SSAB (Borlänge, Sweden) exhibit a martensitic and a mixed microstructure of approx. 60% ferrite and 40% martensite in the delivery condition, while the measured grain size is approx. ASTM 11.5 and ASTM 13, respectively (see Figure 2b,c).

In the initial condition, the tensile strength of the steels 22MnB5 in the hard and soft state, Docol 1400M and DP1000 is 1600 MPa, 450 MPa, 1480 MPa, and 1050 MPa, respectively. Furthermore, the uniform strain is determined to be 3.25%, 18%, 2.75%, and 8%, respectively.

### 2.1. Rapid Austenitization and Quenching

**Figure 3:**
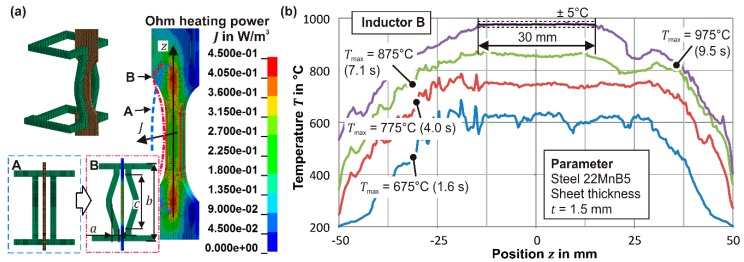
(**a**) Optimized Inductor B; (**b**) Measured temperature along the height during warming up.

To find an ideal austenitization and quenching process for highest strength-strain values, the major samples in the form of tensile specimens are heated to the austenitization temperature *T*_γ_ by induction, held for the dwell time *t*_γ_ and quenched at a varying cooling rate *cr* by air or a water bath. The major specimens possess a parallel length of 30 mm and a width of 12.5 mm. The shoulder has a radius of 20 mm according to DIN EN ISO 50125. The heating is conducted at 400 kHz with a channel inductor powered by an AXIO 10/450 from Huettinger and controlled by a pyrometer METIS M16 from SensorTherm (Sulzbach Ts., Germany). With this system, an average heating rate of 100 K/s is achieved, so that the highest temperature of 1100 °C is reached in 11 s. Figure 3a shows the induction coil (inductor B), which was designed by numerical optimization and ensures a homogeneous temperature along the height. For this, in the first step, the required heat along the height for warming up and covering the convection and radiation losses was calculated analytically and subsequently used as the objective for the optimization in Ls-Opt. 

The optimized oval inductor B is characterized by the indentation with the dimensions *a* = 2.1 mm, *b* = 108 mm, and *c* = 9 mm (see Figure 3a), and, therefore, allows a very homogeneous heating to the target temperature as shown exemplarily in Figure 3b. After the dwell time has elapsed, the cooling via two adjustable air nozzles or the water bath is initiated so that cooling rates *cr* = 30 K/s, 50 K/s (air pressure), and 2200 K/s (water bath) occur. To evaluate the first transformation temperature during cooling, the expansion is monitored by the extensometer PMA-12/V7-1 from Maytec. 

### 2.2. Thermo-Mechanical Testing

Thermo-mechanical tests are conducted for the clocked treatment comparable to the proposed progressive die technology. In accordance with the austenitization and quenching procedure, the temperature controller is used to achieve a defined temperature time curve within the tensile test machine Z250 from Zwick (Ulm, Germany). To cover the main characteristics of the hot forming process, the major specimens are heated by induction, cooled by environment to forming temperature, deformed and subsequently rapid-cooled to an intermediate temperature, held for a specific time, and finally cooled rapidly to room temperature. Here, the deformation is conducted at a constant strain rate of 0.33 s^−1^. Furthermore, a more powerful cooling nozzle using an air-water mixture is implemented to achieve the higher cooling rate *cr* = 150 K/s comparable to tool cooling. During the thermo-mechanical tests, the extension is observed via the contact extensometer Maytec PMA-12/V7-1 (Singen, Germany). To prevent elastic and plastic strain from thermal expansion and transformation induced strain, the sample is mechanically uncoupled via a clutch directly after the plastic deformation. 

### 2.3. Tensile Tests

Tensile tests are conducted according to DIN EN ISO 50125 to find the ultimate tensile strength *TS* and the uniform strain *ε*_u_. Here, the uniform strain is examined to exclude scattering strain at fracture, which is noticeable in particular for brittle material conditions. The produced major specimens are reduced by water jet cutting to minor specimens with a parallel length of 20 mm and inspected on the machine Z250 from Zwick at a cross-beam speed of 0.1 mm/s and a pre-force of 100 N. The specimens are reduced before testing due to a softer microstructure next to the shoulder caused by lower local cooling rates.

### 2.4. Microstructure and Hardness Analysis

The obtained microstructures resulting from different test conditions were analyzed by light optical microscopy (LOM), (Zeiss Axio, Jena, Germany) scanning electron microscopy (SEM), (Tescan LYRA3 XM, Brno, Czech Republic), and hardness measurements. To prepare the samples, a mechanical polishing procedure with 1 μm diamond suspension in the last step is carried out. Finally, the samples were etched with a 3% nitric acid for approx. 2–5 s. The microstructure morphology is observed with a magnification of 200×, 500×, and 1000× with the LOM and up to 10,000× with the SEM. Several hardness values are measured on the polished surface by the Vickers method according to DIN EN ISO 6507-1 with the test force 10 kgf for macroscopic properties and 0.01 kgf for the detection of single phases. The measured values are finally averaged to representative values.

### 2.5. X-Ray Diffraction

To determine the contents of present volume fractions, X-ray diffraction measurements with a Bruker Advance D8 and a copper tube (Billerica, United States) were carried out for selected specimens. The range of 2*θ* was chosen from 30° to 120°. In addition to the typical intensities of lattice direction {1 1 0}, {2 0 0}, and {2 1 1} for the typical body-centered cubic martensite, measured intensities of the direction {2 0 0} and {2 2 0} for the face-centered cubic crystal lattice would indicate amounts of austenite.

## 3. Rapid Austenitization

When the rapid austenitization is applied, the A_c1_ and A_c3_ temperatures increase and, thus, higher temperatures for homogenization are mandatory. Since the transformation is strongly dependent on the microstructure in terms of the chemical composition, the present phases, the precipitated carbides, and the grain size, the prediction of the austenitization becomes inaccurate, especially when high heating rates are applied [15]. For example, the commercial software JMatPro (V9, Sente Software Ltd., United Kingdom) predicts the phase transformation kinetics on the basis of the JMAK equation and specifies a homogenized austenite at 1016 °C, 1011 °C, and 1015 °C for the steels 22MnB5, Docol 1400M, and DP1000, respectively, after heating at 100 K/s with an initially normalized microstructure. For a quenched and tempered microstructure, the temperature of homogeneous austenite decreases to 894 °C, 890 °C, and 898 °C, respectively. However, the following insight demonstrates strongly deviating temperatures for the homogenization and setting of high strength, in particular when mixed phases are present. To determine suitable austenitization parameters, the steels are treated at *T*_γ_ = 950 °C, 1025 °C, and 1100 °C for *t*_γ_ = 3 s and 10 s with a subsequent cooling at *cr* = 30 K/s, 50 K/s, and 2200 K/s. Finally, selected ideal austenitization parameters based on the experimental observation serve as the input for the extended thermo-mechanical analysis. 

### 3.1. Austenite-Martensite Transformation

**Figure 4:**
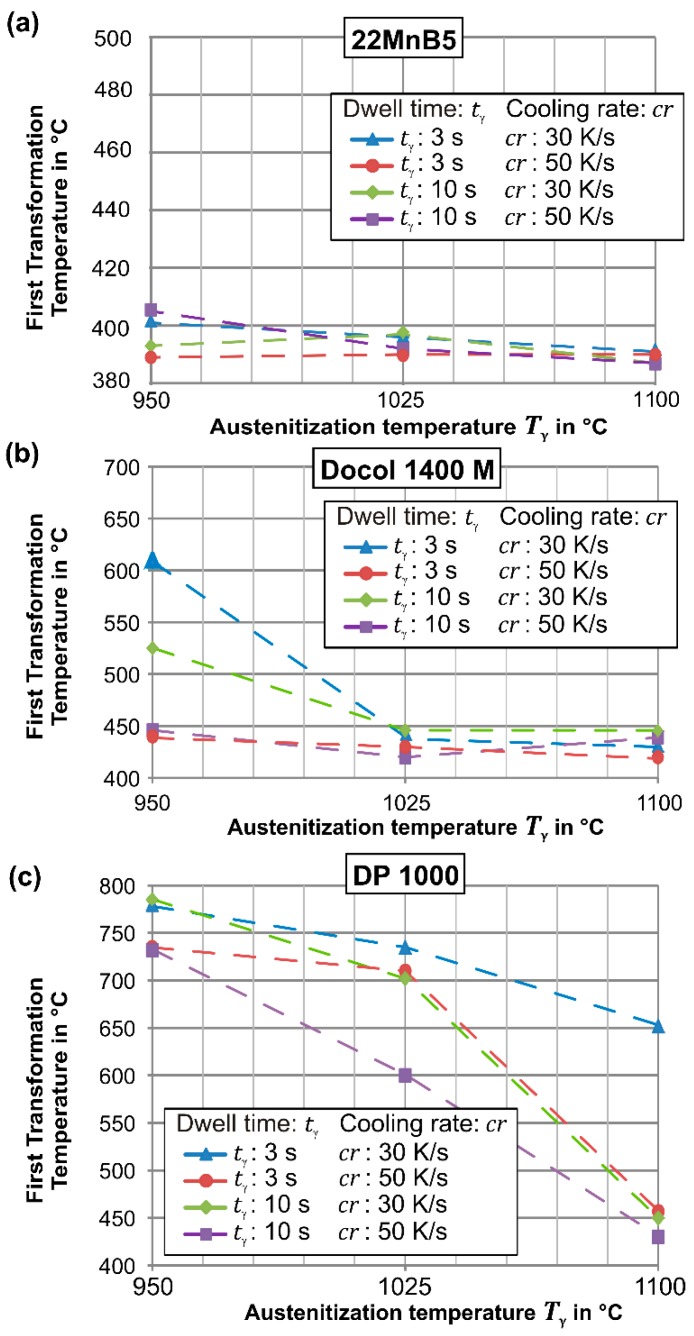
Measured first transformation temperatures during cooling for (**a**) 22MnB5; (**b**) Docol 1400M; and (**c**) DP1000.

The first transformation temperature of the 22MnB5 steel during cooling after the rapid austenitization is shown in Figure 4a. For all temperatures, dwell times, and cooling rates, the transformation occurs below 405 °C so that a fully martensitic microstructure develops. This result is in accordance with Naderi *et al.* [21] and Nikravesh *et al.* [22], who showed that higher temperatures and longer dwell times cause a reduction of the martensite start temperature M_s_ and, moreover, lower cooling rates lead to an increased start temperature. The imaged microstructures indicate that the increasing dwell time especially at 1100 °C cause a huge grain growth. However, a coarser grain structure leads rather to a formation of martensite than a diffusion controlled formation of cementite because of the increasing diffusion length [23]. This mechanism finally requires a higher driving force for the martensite transformation, which is obtained at lower temperatures. Regardless of the temperature, even the short-term treatment at the minimum cooling rate *cr* = 30 K/s is sufficient to format a fully martensitic structure. This observation agrees with the findings by Holzweißig *et al.* [5], who investigated short dwell times and observed outstanding mechanical properties for the rapid processing. Moreover, when the initial microstructure is martensitic, a slightly lower transformation temperature is determined (martensitic structure: A_c3_ = 833 °C *vs.* ferritic-pearlitic structure A_c3_ = 845 °C) and already *T*_γ_ = 950 °C is sufficient for the full hardening. Thus, the 22MnB5 steel possesses in both the soft and hard condition a favorable distribution of elements, which simplifies the rapid austenitization.

**Figure 5:**
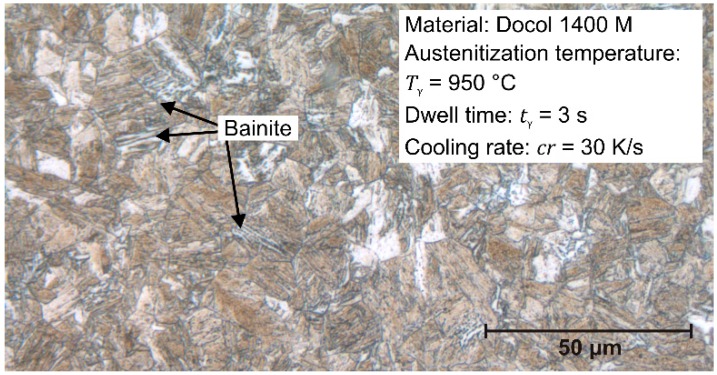
Microstructure of Docol 1400M after austenitization at *T*_γ_ = 950 °C for *t*_γ_ = 3 s, *cr* = 30 K/s.

In contrast to the 22MnB5 steel, the first transformation temperature of the Docol 1400M steel is strongly dependent on the cooling rate. When the austenitization takes place at *T*_γ_ = 950 °C (A_c3_ = 834 °C), the cooling rate *cr* = 30 K/s is not sufficient to transform a fully martensitic microstructure. As Figure 5 depicts, the microstructure exhibits a slight content of bainite. When the cooling rate or the dwell time increases, the first transformation decreases to finally 445 °C so that mainly a martensitic transformation occurs. For higher temperatures, the martensite start decreases to 420 °C, which is caused by the grain growth and the necessary higher driving force. The reason for the cooling rate-dependent phase transformation at *T*_γ_ = 950 °C is an inhomogeneous distribution of carbon, so that a critical concentration allows the diffusion controlled transformation. Thus, higher temperatures and dwell times homogenize the carbon and lead to a diffusionless transformation to martensite.

Furthermore, Figure 4c depicts the first phase transformation temperature of DP1000 steel. For rising temperatures, at each dwell time and cooling rate, a significant reduction of the first transformation temperature becomes obvious. For the lowest temperature *T*_γ_ = 950 °C, all configurations account for an early transformation entrance above 700 °C so that diffusion controlled ferritic and bainitic phases occur. Although the A_c3_ temperature of 868°C was identified during heating, the temperature *T*_γ_ = 950 °C and the cooling rate *cr* = 50 K/s above the critical cooling rate of 45 K/s, which was determined by Naderi [14], are insufficient to enable a martensitic transformation. Only for the highest austenitization temperature *T*_γ_ = 1100°C, the longer dwell time *t*_γ_ = 10 s and higher cooling rate *cr* = 50 K/s are feasible to reach the martensite start and develop a martensitic structure. Further hardness and tensile tests of water bath quenched samples without dilatometry reveal even for *t*_γ_ = 3 s and *T*_γ_ = 950 °C a fully martensitic structure with a high macroscopic hardness of 440HV10 and *TS* = 1407 MPa (see Section 3.2).

**Figure 6:**
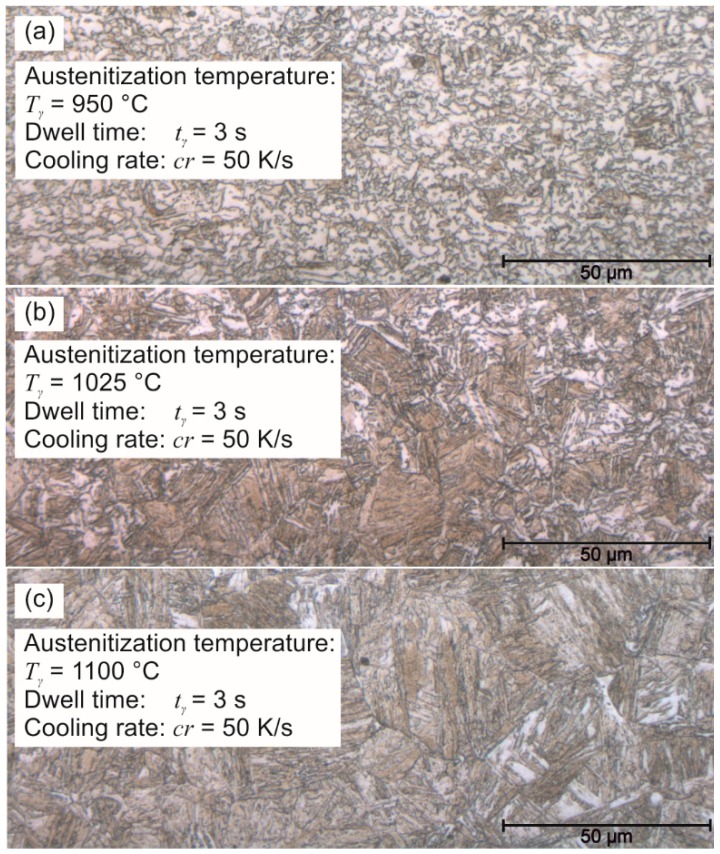
Microstructure evolution of DP1000 for varying austenitization temperature: (**a**) *T*_γ_ = 950 °C; (**b**) *T*_γ_ = 1025 °C; (**c**) *T*_γ_ = 1100 °C.

Thus, the critical cooling rate is the major controlling factor of martensite formation and is not constant but rather decreasing for higher dwell times and temperatures. This phenomenon can be traced back to two reasons: higher temperatures and longer holding times cause a grain growth of approx. a factor of 2 for *T*_γ_ = 1025 °C and a factor of 5 for *T*_γ_ = 1100 °C compared to *T*_γ_ = 950 °C combined with *t*_γ_ = 3 s (see Figure 6a–c). Thus, the diffusion of carbon is reduced and fewer lattice defects are available for the nucleation of carbides so that the diffusionless transformation is favored. Furthermore, short annealing times and low temperatures hinder a homogenization of the carbon content, whereby imbalanced carbon concentrations in the original martensite and ferritic phases with high and low martensite start temperatures occur [24]. Thus, not only a transformation of ferrite and bainite in low-carbon phases is preferred, but also the martensite phases are impoverished of carbon so that a recombination of martensite is impeded. As the LOM reveals, the low cooling rate *cr* = 30 K/s after holding at *T*_γ_ = 1025 °C promote high bainitic fractions. Therefore, the setting of a high martensitic fraction in the low carbon steel DP1000 requires either an accelerated cooling or a certain grain growth through higher austenitization temperatures so that a quenching at lower cooling rates becomes possible. 

### 3.2. Mechanical Properties

**Figure 7:**
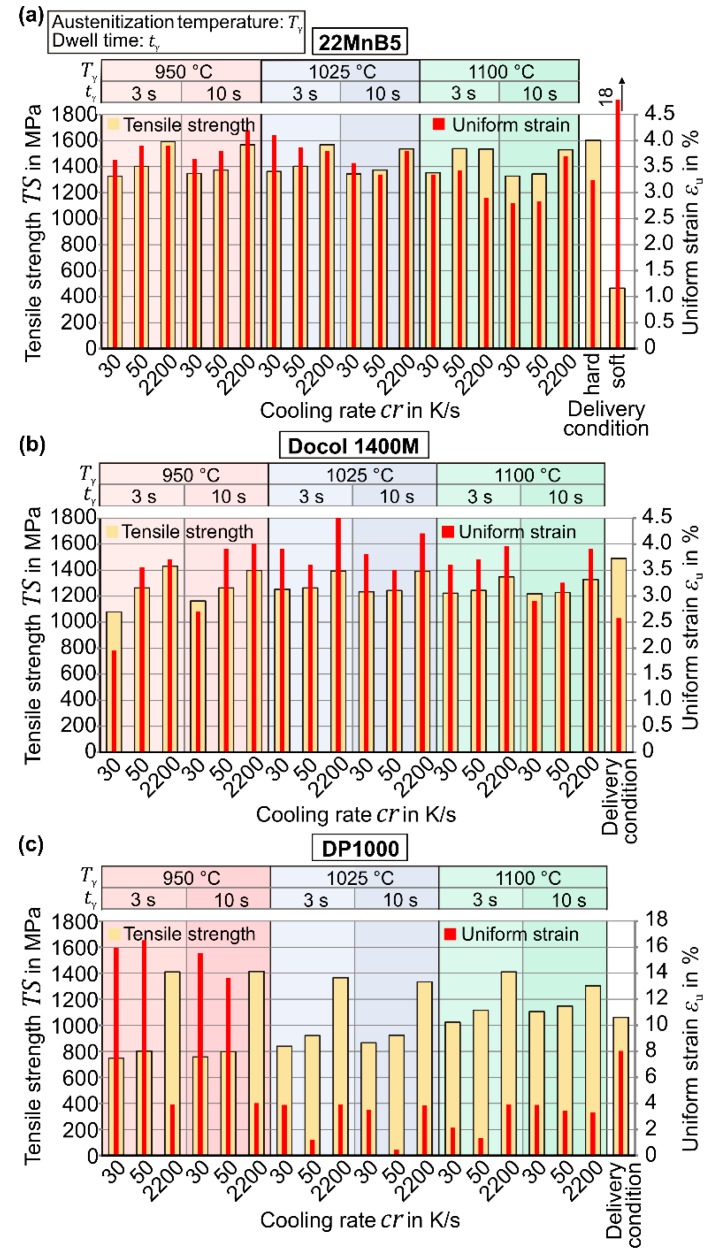
Tensile strength and uniform strain for (**a**) 22MnB5; (**b**) Docol 1400M; and (**c**) DP1000.

Figure 7a depicts the achieved properties of 22MnB5 steel after austenitization. Since a fully martensitic structure is already obtained at *T*_γ_ = 950 °C for *t*_γ_ = 3 s, the maximum tensile strength *TS* = 1590 MPa is reached with the highest cooling rate *cr* = 2200 K/s. For each austenitization temperature and dwell time, rising cooling rates provide higher tensile strength values. This effect is driven by the disorder of the lath structure of martensite, which increases for stronger cooling rates [25]. 

**Figure 8:**
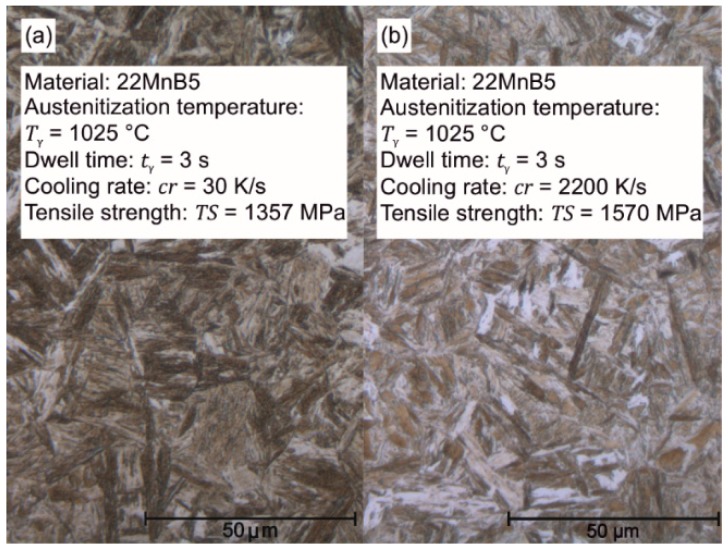
Microstructure of 22MnB5 for alternating cooling rates: (**a**) Air cooling at 30 K/s; (**b**) Water cooling at 2200 K/s.

When the cooling is accelerated, more nucleation points are utilized and cause, thus, a finer and disordered martensitic structure (see Figure 8). When the austenitization temperature and dwell time increase, the tensile strength gradually decreases by 60 MPa (*T*_γ_ = 1100 K/s, *t*_γ_ = 10 s). According to the Hall-Petch equation [26,27], this phenomenon can be explained by the increasing grain size because a finer structure and more grain boundaries impede dislocation motion. The grain growth is caused by the thermal activation, which is delayed in the case of the 22MnB5 steel through alloy elements, e.g., aluminum, titanium, and niobium. Independent of the dwell time and cooling rate, the short austenitization with temperatures up to *T*_γ_ = 1025 °C allows higher uniform strains (compare with delivery condition: *ε*_u_ = 3.24%), reaching the maximum *ε*_u_ = 4.2% for *T*_γ_ = 950 °C, *t*_γ_ = 10 s, and *cr* = 2200 K/s. Holzweißig *et al.* [5] assume that this effect is induced by the initially finer microstructure and retained carbides because more defects are used for the nucleation and later grain refinement.

Similarly, the Docol 1400M steel exhibits the highest tensile strength (see Figure 7b) and hardness for each configuration when the highest cooling rate is used. Since the combination of *T*_γ_ = 950 °C, *t*_γ_ = 3 s, and cooling rate *cr* = 30 K/s enforces a slight bainitic fraction, not only the corresponding tensile strength is unfavorably low (*TS* = 1075 MPa) but also the small uniform strain indicates a brittle microstructure (*ε*_u_ = 2%). When higher cooling rates are applied, not only a bainitic fraction is prevented and the strength increases, but also the ductility is improved drastically (*cr* = 2200 K/s: *TS* = 1430 MPa, *ε*_u_ = 3.7%). When the temperature and dwell time increase slightly, the maximum cooling rate *cr* = 2200 K/s entails maximum uniform strain (*ε*_u_ = 4.5% for *T*_γ_ = 1025 °C and *t*_γ_ = 10 s), while the strength remains at a constant high level of approx. *TS* = 1400 MPa. In the case of the Docol 1400M steel, the effect of grain growth for high austenitization temperatures is visibly high. Compared to the delivery condition, an austenitization for *t*_γ_ = 3 s and later cooling at *cr* = 50 K/s increases the grain size approx. at *T*_γ_ = 950 °C by the factor 1.5, at *T*_γ_ = 1025 °C by the factor 2.5, and at *T*_γ_ = 1100 °C by a factor of three. Thus, the rapid austenitization and quenching enables not only an equivalently high strength, but also the ductility can be improved significantly (delivery condition: *ε*_u_ = 2.5%).

Similarly, also the DP1000 steel manifests the highest strength for the high cooling rate combined with *T*_γ_ = 950 °C. At higher temperatures, lower cooling rates finally lead to a fully martensitic structure, but the increasing grain size limits the strength significantly (see Section 3.1). Particularly the condition after austenitization at *T*_γ_ = 950 °C reflects the complex behavior of the initial dual phase steel. Both configurations, medium strength combined with high ductility and high strength combined with a moderate ductility can be achieved. This phenomenon is caused by the interaction of low and high carbon concentrations and vanishes due to homogenization at high temperatures. 

### 3.3. Parameter Configuration for High Strength

**Figure 9:**
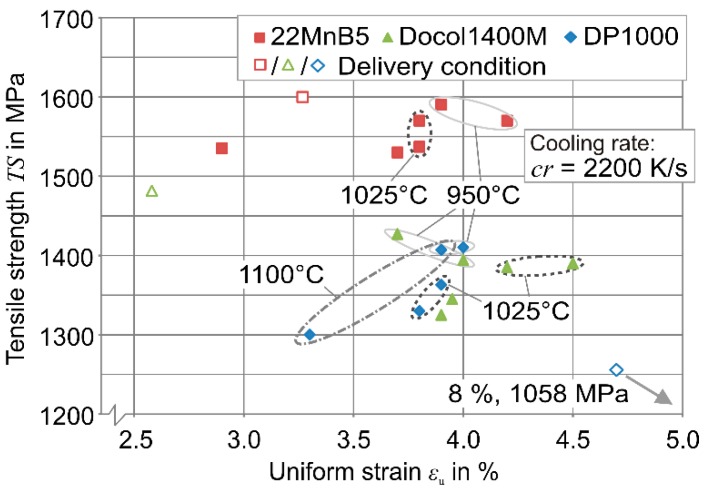
Tensile strength and uniform strain after short austenitization and water bath quenching.

The brief austenitization study for the high strength steels 22MnB5, Docol 1400M, and DP1000 reveal that high-strength values can generally be found with short-term austenitization at low to medium overheating above the equilibrium A_c3_ temperature. Figure 9 depicts the highest tensile strengths over the uniform strain for *cr* = 2200 K/s. For each steel, the ideal strength is achieved by austenitization at *T*_γ_ = 950 °C. To obtain the highest strength also at lower process relevant cooling rates, the dependency on the critical cooling rate has to be taken into account. Thus, for the dual phase steel, higher temperatures are advantageous despite the increasing grain growth because, after a homogenization of the carbon concentration, even lower cooling rates are sufficient for a fully martensitic transformation. 

Similarly, the Docol 1400M steel manifests a significant bainitic fraction after quenching from *T*_γ_ = 950 °C at *cr* = 30 K/s so that a higher austenitization temperature is necessary. Only the 22MnB5 steel exhibits an adequate austenite microstructure after short heating at relatively low temperatures through the microalloyed elements so that a higher driving force is not necessary. For the subsequent investigation of the microstructure evolution under a thermal cycle, in relation to the progressive die process, the austenitization parameters for the 22MnB5, the Docol 1400M, and the DP 1000 steels are chosen to be *T*_γ_ = 975 °C, 1025 °C, 1075 °C, and *t*_γ_ = 5 s, 5 s and 3 s, respectively.

## 4. Intermittent Quenching

**Figure 10:**
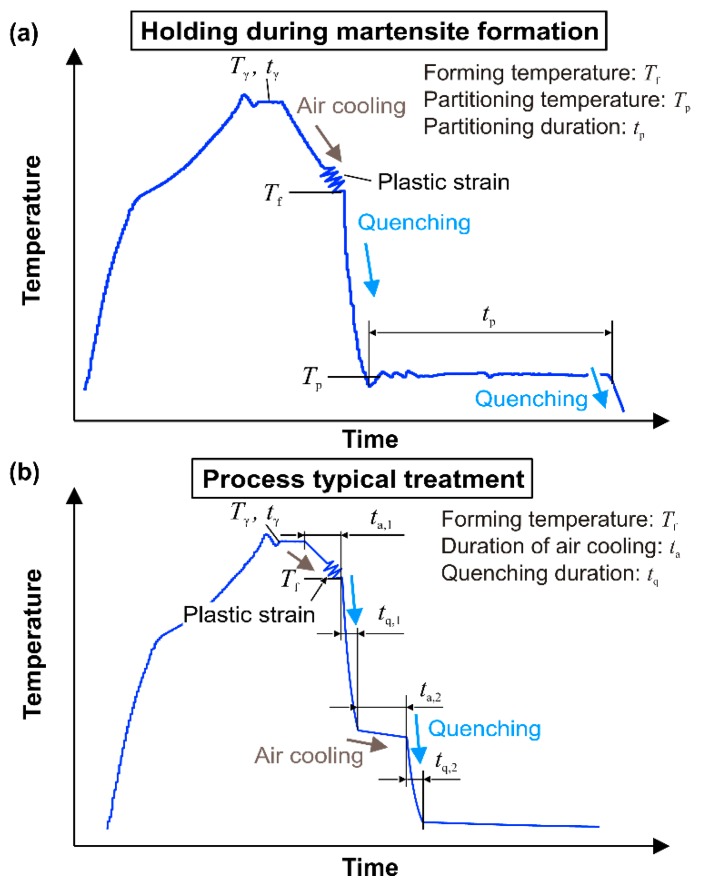
Temperature time curves: (**a**) Treatment in Section 4.1; and (**b**) treatment in Section 4.2.

The intermittent quenching after austenitization is typical of the proposed technology, whereby the heat is removed by multiple stages so that different temperature time curves combined with continuous cooling and isothermal holding are studied. According to chapter 2, the temperature profile is obtained by induction heating and holding, air cooling, forming and spray quenching, a second phase of holding, and, finally, accelerated cooling to room temperature. Figure 10 illustrates the representative temperature profile and settling parameters, which are varied to analyze the effect of holding during martensite formation, as well as achievable microstructures after continuous cooling. 

### 4.1. Holding during Martensite Formation

In its original sense, in the quenching and partitioning process the carbon diffuses into austenite during the holding so that a further transformation to martensite is hindered during cooling to room temperature. Comparably to the TRIP steels, the retained austenite allows more work hardening and a clearly improved ductility when plastic deformation is applied [28]. Generally, after austenitization, a quenching stage to temperatures between martensite start and finish point is applied, which is followed by a warmer partitioning stage so that the rate of carbon diffusion increases but the separation of carbides is not promoted. In addition to this procedure, Liu *et al.* [29] processed silicon enriched press-hardenable steels by austenitization and direct partitioning without an interposed quenching so that volume fractions of retained austenite of up to 18.5% were achieved. Here, the partitioning time amounts to only a few seconds in contrast to the long dwell time of up to several minutes of conventional Q and P processing [30]. Thus, the upset of an enhanced microstructure in the three selected steels is analyzed through a partitioning stage directly after quenching at a temperature between martensite start and finish point. For each steel, two dwell temperatures *T*_p_ are selected and held for the dwell times *t*_p_ = 10 s and 30 s (see Table 2). According to Figure 10a, each configuration is carried out without and with a total strain *ε* = 20% at *T*_f_ = 800 °C directly before quenching to the intermediate temperature.

**Figure 11:**
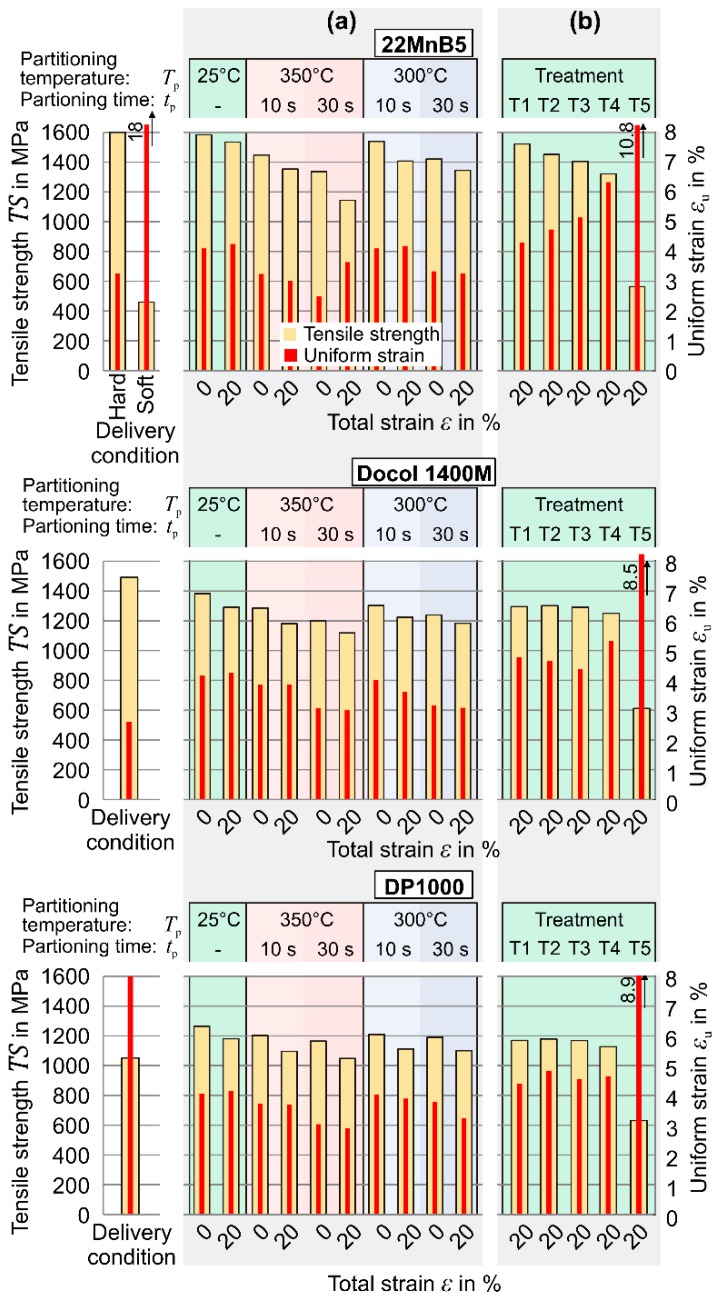
Tensile strength and uniform strain after quenching: (**a**) with holding during martensite formation; and (**b**) with process typical treatment.

Figure 11a depicts the mechanical properties of the processed samples. Regardless of the chemical composition, the highest tensile strength (22MnB5: *TS* = 1583 MPa, Docol 1400M: *TS* = 1385 MPa, DP 1000: *TS* = 1263 MPa) is achieved for direct quenching to room temperature without deformation. The additional deformation enhances the ductility slightly but leads to a reduced strength. Due to the rising dislocation density, a higher driving force for conversion into martensite is required so that higher cooling rates become necessary for an equivalent transformation [31]. In contrast to the enhanced toughness through a more complex structure, the isothermal holding during martensite conversion is accompanied by both decreasing strength and uniform strain. This trend develops monotonically for both increasing dwell time and higher dwell temperatures. 

**Figure 12:**
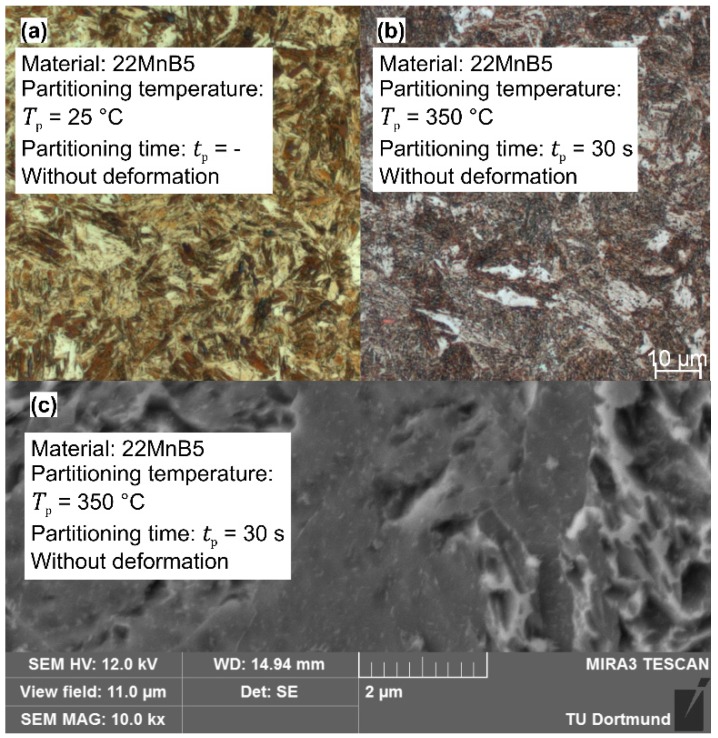
Microstructure of 22MnB5: (**a**) LOM of quenching to room temperature; (**b**) LOM of quenching to 350 °C and holding for 30 s; and (**c**) SEM: Formation of carbides through holding procedure.

**Figure 13:**
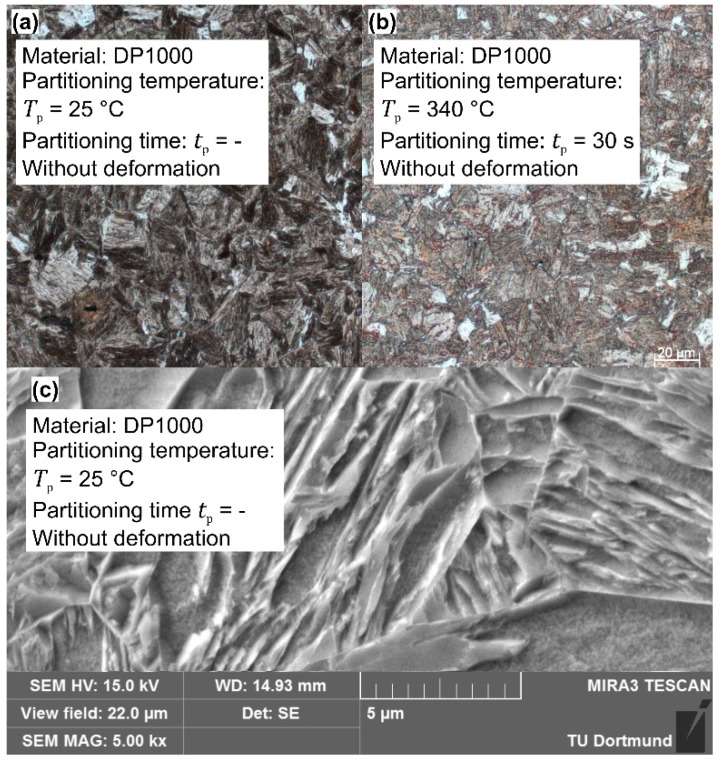
Microstructure of DP1000: (**a**) LOM of quenching to room temperature; (**b**) LOM of quenching to 350 °C and holding for 30 s; and (**c**) SEM of quenching to room temperature.

In particular the LOM and SEM measurements of 22MnB5 steel samples with deformation illustrate an indistinct martensitic structure when the warmer temperature *T*_p_ = 350 °C is applied for *t*_p_ = 30 s (see Figure 12). Here, the reduced tensile strength is probably attributable to the rising amount of bainite. Micro Vickers measurements indicate that for the long duration of *t*_p_ = 30 s at *T*_p_ = 350 °C, a formation of bainite occurs because the amount of bright fractions is more pronounced and has a lower hardness (bright fractions: < 480 HV0.01 compared to martensitic fractions >600 HV0.01). Furthermore, SEM pictures at high magnification of partitioned samples show a structure of fine white particles, which suggests an excretion of carbides (see Figure 12c). Additionally, the analysis of the Docol 1400M and DP1000 steels indicate a martensitic structure combined with volume fractions of bainite when the partitioning time and temperature rise. Figure 13 depicts the microstructure of DP1000 steel, which manifests a rising content of bainite-similar volume fractions when the indirect cooling method is applied. Similarly to the 22MnB5 steel, the micro hardness measurements confirm a lower hardness in the bright areas after holding at *T*_p_ = 340°C (< 490 HV0.01). In contrast to the previous steel, the bright areas possess, also after direct quenching, a lower hardness and the additional holding causes rather a propagation of the soft zones. In the case of Docol 1400M steel, the direct quenching method leads to intermediate hardness in the bright areas (~515 HV0.01), and, after the intermittent cooling, the same effect as in the other steels occurs in a more pronounced manner (<480 HV0.01 for *t*_p_ = 30 s at *T*_p_ = 340 °C, without deformation). 

**Figure 14:**
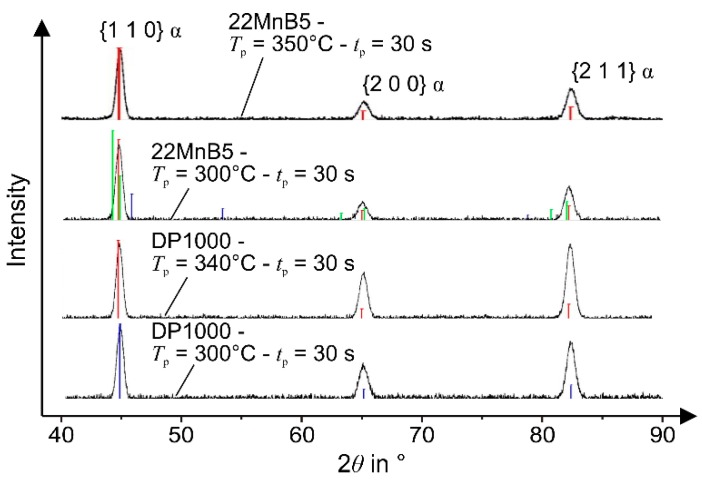
X-ray detection of processed samples without deformation.

Figure 14 shows the X-ray measurement of the 22MnB5 and DP1000 steels after holding for *t*_p_ = 30 s and at different dwell temperatures. None of the selected configurations show an austenite peak at the lattice spacing of the direction {2 0 0} or {2 2 0}. Speer *et al.* [28] establish that the stabilization of austenite is reached by a higher carbon concentration, which is induced by the carbon leaving the martensite. This phenomenon is hindered by bainite and carbide formation so that, as a consequence of the insufficient stabilization, further martensitic and ferritic phases appear. The formation of cementite can be decreased by the addition of silicon or other elements as aluminum and phosphorus. As shown by Santofimia [32] or by Kim *et al.* [33], with low silicon steels (Si: 0.35 and 0.32 wt.%, respectively), austenite is also retained while aluminum ensures the inhibition of carbides. Thus, the conventional steels are not suitable for setting austenite fractions through an intermittent cooling procedure. In this context, the etched ferrite in Figure 12c suggests the decomposition of austenite to ferrite with the precipitation of fine carbides next to the grain boundaries. 

### 4.2. Intermittent Process Typical Cooling Cycles

In addition to cooling with holding during martensite formation, the effect of continuous cooling during the martensite conversion is analyzed for example process designs. As before, the austenitization temperature *T*_γ_ and dwell time *t*_γ_ are configured according to Section 3.3. To obtain tailored properties, also the feasibility of adjusting soft zones with ferrite or lamellar bainitic fractions through long holding periods before quenching is studied. As shown in Table 3 and Figure 10b, five process typical treatments with a fictive alternating process speed (clock rate *f*_s_) and tool design (blank stages, see Figure 1a) are applied. In order to reach a maximum or minimum heat release and cause either hard martensitic or soft ferritic microstructures, the duration of quenching *t*_q_, which corresponds to the resting of the punch in the lower tool position, is varied. 

**Figure 15:**
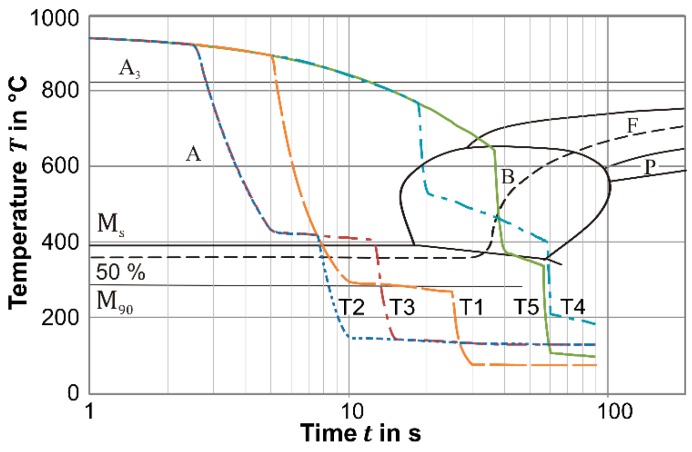
Continuous time temperature chart of 22MnB5 and configured process typical treatments.

From the received mechanical properties in Figure 11b, in particular the steel 22MnB5 stands out with a high tensile strength (1522 MPa) in combination with a satisfying strain (*ε*_u_ = 4.3%) after the treatment T1. This treatment without a blank stage between the heating and forming stage has a pause of approx. 5 s after austenitization before the quenching starts and, thus, allows a direct cooling to the M_90_ temperature so that the diffusionless transformation is almost completed (see Figure 15). When the higher clock rate of *f*_s_ = 12 min^−1^ is applied (T2 and T3), the primary quenching over *t*_q_ = 2.5 s reduces the temperature to approx. 420 °C. This resting phase directly above martensite start point accelerates the kinetic compensation of disequilibrium and causes a lack of carbon in the austenite. Finally, the intervention to speed up the process with shorter quenching periods leads to lower strength but higher strain (T3 *vs.* T1: *TS* decreased by 118 MPa, *ε*_u_ increased by 0.8%). When very long resting periods are applied, the strength decreases dramatically (T4 and T5). Here, the slow cooling allows the diffusion-controlled formation of bainitic and full ferritic structures, by which also soft material conditions in terms of tailored properties can be achieved.

Similarly, also for the Docol 1400M and DP1000 steels, a high tensile strength with an adequate uniform strain can be achieved when the process typical treatment with a medium to high clock rate (*f*_s_ = 6–12 min^−1^) is applied (approx. *TS* = 1250 and 1150 MPa, respectively). As Figure 11b indicates, for the boron-free steels, the achieved strength is reduced when the process speed decreases (lower clock number *f*_s_ and additional blank stage). When the clock rate decreases to the minimum of *f*_s_ = 3 min^−1^, the microstructure in both steels becomes fully ferritic and the tensile strength is reduced to approx. 600 MPa (T5). Since both boron-free steels contain a higher amount of silicon and also the grain structure is coarsed by the high austenitization temperature, the ductility of the 22MnB5 steels in the soft state is superior (*ε*_u_ = 10.8% *vs.* 8.5% and 8.9%, respectively).

### 4.3. Correlation of Mechanical Properties 

**Figure 16:**
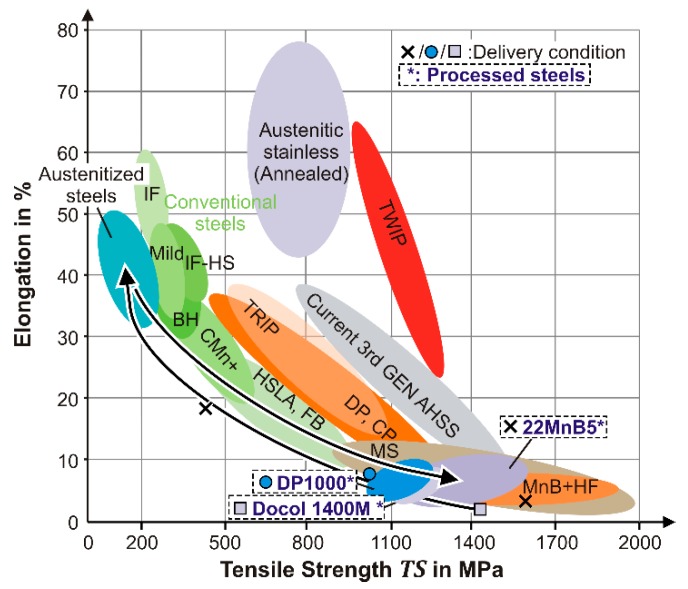
Classification of achieved properties in relation to standard steels according to Keeler and Kimchi [35].

The brief introduction of possible process settings indicate that a wide range of tailored properties can be covered by the proposed hot forming process. As shown in Figure 16, comparable to the conventional hot stamping process, the formability is dramatically enhanced through the austenitization. Although, in the fast clocked process, only a short-term quenching is possible, mechanical properties can be achieved which exceed the delivery condition in terms of strength and/or strain. Finally, the treatment on the basis of the determined austenitization parameters and alternating cooling conditions covers a wide range of high strength values from 1000 MPa to 1600 MPa for the three selected steels (see Figure 16). According to Section 3.2, also a significantly enhanced elongation at fracture is feasible when a lower austenitization temperature and a shorter dwell time are applied. In particular, steels as the DP1000 without grain growth inhibitors and low carbon content require a rapid and accurate heating to avoid a harmful grain coarsening. 

**Figure 17:**
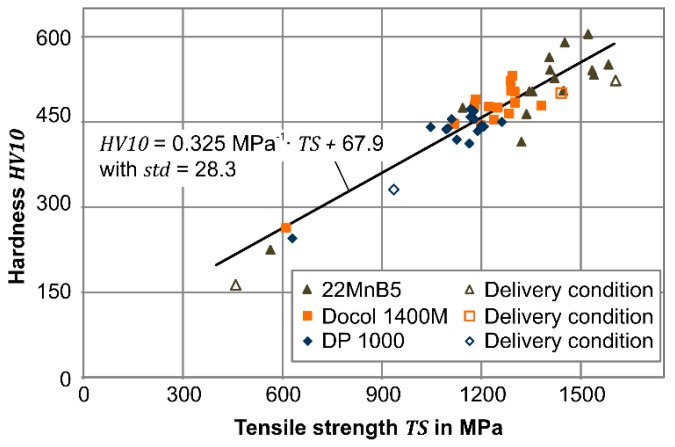
General correlation of measured Vickers hardness and tensile strength for studied steels.

Related to the tensile strength, the achieved macroscopic hardness of a primary martensitic structure differs from a minimum of 410 HV10 for the DP1000 steel to a maximum of 605 HV10 for the 22MnB5 steel (see Figure 17). In addition to the hardness conditions, an expansion to ferritic microstructures (200 HV10) is feasible for all steels under the process relevant parameters. As shown in Figure 17, the hardness and tensile strength develop a linear connection for the whole parameter range comparable to mild steels in different conditions [34]. In terms of the control of the mechanical properties in the rapid hot forming process, a reliable relationship to adapt the tensile strength is given.

## 5. Conclusions

This study reveals a new methodology for an industrially-feasible rapid hot forming process based on a high power and process integrated heating method. The approach allows the flexible setting of tailored properties through both a locally-adapted temperature history from heating to cooling and the triggering of dynamic effects during the phase transformation with the consequence of unique mechanical properties. The results can be used for designing process parameters and offer important hints for obtainable properties of further potential steel grades.

For the investigated high strength steels a good applicability is observed so that tailored properties are achieved with either an improved strength or ductility compared to the conventionally-expected outcome. Furthermore, the process typical intermitted cooling opens various opportunities in terms of tempering or partitioning processes. For example, the processing of the 22MnB5 steel with heating at 100 K/s, a few seconds dwell time and quenching at 150 K/s allows an increased uniform strain by 31% through the additional holding at the M_90_ temperature, whereas the tensile strength is reduced by only 9%. Moreover, the treatment of the annealed steels allows the flexible adjustment of mechanical properties by setting the amount of martensite, bainite, and ferrite. For example, the ductility of the Docol 1400M steel can be increased by 75% when a slightly reduced tensile strength (*TS* = 1300 MPa) is acceptable. Furthermore, the DP1000 steel can be transformed into a ductile condition (*ε*_u_ = 16%, *TS* = 800 MPa) when a low austenitization temperature is selected or into a hard condition (*ε*_u_ = 4.1%, *TS* = 1260 MPa) by applying a high austenitization temperature and intended grain growth. 

Although the austenitization was conducted at a heating rate of 100 K/s, a refinement of primary austenite was not noticed. Since other studies on the austenitization at heating rates of up to 400 K/s revealed a refined structure with improved mechanical properties [5,15,18], the potential of the proposed process is not fully exploited. Furthermore, the X-ray measurement reveals clearly that the partitioning at different temperatures between the martensite starting and finishing temperature is not feasible to retain austenite in the low carbon and low silicon steels. 

Thus, in further studies, the development of retained austenite through an aligned chemical composition (e.g., additional silicon or aluminum) on the basis of this unique process should be investigated. In the best case, the formation of carbides is strongly inhibited so that a grain refinement is also obtained by higher heating rates is allowed. Combined with the proposed technological implementation, these two effects would promote outstanding tailored properties in the large scale production based on progressive dies.

## Figures and Tables

**Table 1:** Chemical composition (wt.%): 22MnB5 [5], Docol 1400M [20], DP1000 [14], * max values.

	C	Si	Mn	P	S	Al	Nb	Cr	B	N	Ni	Mo	Nb + Ti
22MnB5	0.21	0.28	1.18	0.015	0.001	0.030	0.0032	0.18	0.0028	0.0064	0.01	0.02	–
Docol 1400M *	0.20	0.40	1.60	0.020	0.010	0.015	–	–	–	–	–	–	0.10
DP1000	0.15	0.50	1.50	0.010	0.002	0.040	0.015	–	–	–	–	–	–

**Table 2:** Martensite start M_s_ and martensite 90% temperature M_90_ derived from JMatPro and selected partitioning temperatures *T*_p_.

Grade	M_s_ in °C	M_90_ in °C	*T*_p_ in °C
22MnB5	397	282	350; 300
Docol 1400M	380	271	340; 300
DP 1000	379	268	340; 300

**Table 3:** Configured process typical treatments: duration of quenching, tool design, and clock rate.

Treatment No.	Duration of Quenching *t*_q_ in s	Tool Design (Blank Stages)	Clock rate *f*_s_ in min^−1^ (Process Speed)
T1	5	After primary quenching	6
T2	2.5	None	12
T3	2.5	After primary quenching	12
T4	1.5	After primary quenching	3
T5	3.5	After austenitization	3
